# Characterization of the Role of Host Cellular Factor Histone Deacetylase 10 during HIV-1 Replication

**DOI:** 10.3390/v12010028

**Published:** 2019-12-26

**Authors:** Xiaozhuo Ran, Zhujun Ao, Titus Olukitibi, Xiaojian Yao

**Affiliations:** Laboratory of Molecular Human Retrovirology, Department of Medical Microbiology, Max Rady College of Medicine, Rady Faculty of Health Sciences, University of Manitoba, Winnipeg, MB R3E 0J9, Canada; ranx@myumanitoba.ca (X.R.); Zhujun.Ao@umanitoba.ca (Z.A.); olukitit@myumanitoba.ca (T.O.)

**Keywords:** histone deacetylase 10, HIV-1 integrase, protein interaction, progeny virus infectivity

## Abstract

To date, a series of histone deacetylases have been documented to restrict HIV-1 replication at different steps. In this study, we identified histone deacetylase 10 (HDAC10) as an inhibitory factor against HIV-1 replication. Our results showed that endogenous HDAC10 is downregulated at the transcriptional level during HIV-1 replication. By knocking down HDAC10 in CD4+ T cells with specific shRNAs, we observed that the downregulation of HDAC10 significantly facilitates viral replication. Moreover, RQ-PCR analysis revealed that the downregulation of HDAC10 increased viral integrated DNA. Further, we identified that HDAC10 interacts with the HIV-1 integrase (IN) and that the region of residues from 55 to 165 in the catalytic domain of IN is required for HDAC10 binding. Interestingly, we found that the interaction between HDAC10 and IN specifically decreases the interaction between IN and cellular protein lens epithelium-derived growth factor (LEDGF/p75), which consequently leads to the inhibition of viral integration. In addition, we have investigated the role of HDAC10 in the late stage of viral replication by detecting the infectiousness of progeny virus produced from HDAC10 knockdown cells or HDAC10 overexpressing cells and revealed that the progeny virus infectivity is increased in the HDAC10 downregulated cells, but decreased in the HDAC10 overexpressed cells. Overall, these findings provide evidence that HDAC10 acts as a cellular inhibitory factor at the early and late stages of HIV-1 replication.

## 1. Introduction

Histone deacetylases (HDACs) are enzymes that catalyze the removal of acetyl groups from lysine residues in histones and various non-histone proteins. HDACs are involved in the inhibition of gene expression and a series of HDAC family members have been reported to act as inhibitory factors against HIV-1 infection [1]. For instance, HDAC1 and HDAC2 induce the deacetylation of histone H3 and H4 to regulate the degree of compaction of chromatin, which inhibits viral transcription and subsequently contributes to the formation of HIV-1 latency [2]. Moreover, HDAC6 has been reported to inhibit HIV-mediated target cell fusion and entry by preventing the acetylation of cortical tubulin and reduce progeny virus infectiousness by preventing Vif-mediated A3G degradation [3,4]. Recently, our studies revealed that another member of the HDAC family, Histone Deacetylases 10 (HDAC10), was downregulated by HIV-1 virion-associated envelope glycoprotein in the HIV-infected cell line J-Lat 6.3 [5]. HDAC10 is a member of the HDAC class II B family [6]. HDAC10 consists of 669 amino acid residues with a bipartite modular structure, which comprises an N-terminal Hda1p-related putative deacetylase domain and a C-terminal leucine-rich domain [6,7]. HDAC10 is located in the cytoplasm due to its leucine-rich domain, while it may also enter the nucleus through passive diffusion or in association with nuclear proteins [6]. HDAC10 has been reported to play multiple functions in biological processes, such as the mediation of homologous recombination, autophagy-mediated cell survival, and the cell cycle, depending on its deacetylase activity [8,9,10], while this protein also regulates cellular transcription and cell proliferation, independent of its deacetylase activity [11,12]. In addition to mediating cellular processes, HDAC10 has been reported to participate in viral replication [13]. For instance, HDAC10 has been demonstrated to interact with human cytomegalovirus immediate-early protein 1 (HCV-IE1), a viral factor that is important for viral gene expression at the early stage of viral replication [13]. However, whether HDAC10 plays an important role in HIV-1 replication and what role it plays during HIV-1 replication remains unknown.

HIV-1 integrase (IN) is a viral enzyme that mainly catalyzes the insertion of the viral genome into host chromatin [14]. IN contains three functional domains, including an N-terminal core domain (NTD, residues 1–49), a catalytic core domain (CCD, residues 50–212), and a C-terminal core domain (CTD, residues 213–288) [15]. Accumulating shreds of evidence suggest that HIV-1 IN recruits a series of cellular factors by different domains and catalyzes the insertion of newly reverse-transcribed viral DNA into the host genome [16]. At present, several cellular factors have been identified to interact with the HIV-1 IN, including barrier-to-autointegration factor (BAF), high mobility group protein A1 (HMGA1), integrase interactor 1 protein (INI1/hSNF5), lens epithelium-derived growth factor (LEDGF/p75), polycomb group embryonic ectoderm development (EED) protein, histidine-rich protein 2 (HRP2), heat-shock protein 60 (HSP60), Ku autoantigen 70 kDa (Ku70), and the p300 acetyltransferase (P300) [17,18]. The interaction of HIV-1 IN with these cellular factors is essential for the stability and catalytic activity of HIV-1 IN, which ensures efficient integration [19]. For instance, HSP60 interacts with HIV-1 IN and stimulates the processing and joining activities of IN, which protects this enzyme from thermal denaturation [20]. In addition to catalyzing the insertion of viral DNA into host chromatin, HIV-1 IN is also involved in the morphology and infectiousness of progeny virus [21]. Previous mutagenesis analyses have shown that some mutations in HIV-1 IN not only cause aberrant viral particle morphology, but also lead the progeny virus to become defective by impairing subsequent reverse transcription and integration in incoming target cells [22,23,24,25]. In addition, the disruption of the interaction between HIV-1 IN and cellular factors also results in a replication deficiency of progeny virus due to abnormal HIV-1 IN multimerization [26]. This evidence has collectively indicated that HIV-1 IN is important for both early and late stages of viral replication. However, whether HDAC10 can interact with HIV-1 IN and influence viral integration or progeny virus infectiousness remains unclear.

In this study, our results revealed that endogenous HDAC10 is drastically downregulated in CD4^+^ T cells during HIV-1 replication. To investigate the impact of HDAC10 on HIV-1 replication, we knocked down endogenous HDAC10 in CD4^+^ T cell lines and observed that the downregulation of HDAC10 enhances viral replication by promoting viral integration. Using a co-immunoprecipitation (co-IP) assay, we demonstrated that HDAC10 can specifically interact with HIV-1 IN by binding a region (amino acids 55 to 165) in the core domain of IN. Interestingly, the results also revealed that HDAC10 binding to HIV-1 IN decreases the interaction between LEDGF/p75 and IN. Moreover, we detected the infectiousness of the progeny virus from HDAC10 knockdown or overexpressing cells and found that the downregulation of HDAC10 enhances while the overexpression of HDAC10 decreases progeny virus infectiousness. Thus, HDAC10 can inhibit viral replication by affecting viral integration and progeny virus infectiousness.

## 2. Materials and Methods

### 2.1. Plasmid, Chemicals, and Antibodies

HIV-1 proviral plasmid pNL4.3, HIV-packaging plasmids pCMVΔ8.2 and CMV-VSV-G were described previously [27,28,29,30]. HDAC10 shRNA-1 (target: 5′-ATCCCATCTAAGAGGTACAGG-3′) and HDAC10 shRNA-2 (target: 5′-TGCGGTGTCATTTCTGCGGTG-3′) lentiviral plasmid and Scramble control plasmid (target: 5′-CCTAAGGTTAAGTCGCCCTCGCTCGAGCGAGGGCGACTTAACCTTAGG-3′) were purchased from Open Biosystem. HDAC6-FLAG expression plasmid was purchased from Addgene [31]. HDAC10-Myc expression plasmid was purchased from Sino Biological Company. pFLAG-HDAC10 H135A was kindly provided by Dr. Li Y [10]. The green fluorescent protein-integrase (GFP-IN) wild type fusion protein, GFP-IN mutants, and IN-YFP were described previously [32,33].

The primary antibodies were used for western blot, including rabbit anti-GFP polyclonal antibody (purchased from Molecular Probes), rabbit anti-HDAC6 (purchased from Santa Cruz, CA, USA), anti-T7-HRP conjugated antibody (purchased from Novagen, WI, USA), mouse anti-Tubulin antibody (purchased from Sigma, St Louis, MO, USA), mouse anti-HDAC10 antibody (purchased from Santa Cruz, CA, USA), and rabbit monoclonal antibodies against acetyl- group in lysine protein (Abcam, Cambridge, UK). HIV-1 p24 monoclonal antibody was used in western blot and ELISA, which was described in our previous publication [34]. HRP-conjugated donkey anti-rabbit IgG and sheep anti-mouse IgG were used as the secondary antibody, which were purchased from Amersham Biosciences (Mississauga, ON, Canada).

### 2.2. Cell Culture and Primary Cell Isolation

Human embryonic kidney 293T, TZM-b1 were cultured in Dulbecco’s modified Eagle’s medium (DMEM), and Jurkat and C8166 T cells were cultured in RPMI-1640 medium. These mediums were supplemented with 10% fetal bovine serum (FBS) as well as 100 unit/mL penicillin and 100 μg/mL streptomycin. Human peripheral blood mononuclear cells (PBMCs) were isolated from the blood of healthy adult volunteers and isolated by sedimentation in Ficoll-Hypaque (purchased from Sigma-Aldrich). CD4^+^ T lymphocytes were isolated from PBMCs by an EasyStep Human CD4^+^CD25^+^ T Cell Isolation Kit (purchased from Stemcell Technologies). Purified unstimulated CD4^+^ T cells and PBMCs were cultured in RPMI-1640 medium, which was supplemented with 10% FBS, penicillin (100 unit/mL), streptomycin (100 μg/mL), and IL-2 (10 U/mL).

### 2.3. Generation of Virus and Infection

HIV-1 (N119 strain, Cat# 1392) was obtained from the national institutes of health (NIH) AIDS research and reference reagent program. Different pNL4.3 viruses were generated by co-transfecting pNL4.3 expression plasmid with GFP or HDAC10 or HDAC10 H135A plasmid. Then, supernatants were collected after 48 h of transfection and subsequently spun at 3000 rpm for 30 min to remove cell debris. The virus was concentrated by ultracentrifugation, which lasted for 90 min at the speed of 35,000 rpm. The concentration of virus or VLP stocks were further quantified by HIV-1 p24 level. For HIV (N119) infection in C8166 and Jurkat cells (or transduced Jurkat cells), which are susceptible to HIV infection, we infected cells with 1 ng or 5 ng of HIV-1 (MOI of 0.1 or 0.5). For primary CD4^+^ T cells, which are relatively resistant to HIV infection, we infected cells with 10 ng of N119 virus (MOI of 1). Different cells were infected with virus for overnight and washed twice with PBS. Cells were collected at indicated time points for further analysis.

### 2.4. Establishment of HDAC10 Knockdown Cell Line

Lentiviral particles were produced by co-transfecting 293T cells with VSV-G expression plasmid, pCMVΔ8.2, and lentiviral vector TRC-Scramble shRNA or TRC HDAC10 shRNA-1 or TRC HDAC10 shRNA-2, respectively. Lentiviral particles were purified by ultracentrifugation from the supernatant. Jurkat cells were transduced with HDAC10 shRNAs or scramble shRNA for 48 h and selected with puromycin (2 μg/mL) After 48 h of selection, cells were harvested and the endogenous HDAC10 level was detected by western blots and normalized by host protein Tubulin.

### 2.5. Water-Soluble Tetrazolium Salt (WST-1) Assay

5 × 10^4^ cells were plated into a 96 well plate and cultured in a final volume of 100 μL/well culture medium in a humidified atmosphere. Cell viability reagent WST-1 10 μL was added into each well at different time points and incubated at 37 °C for 4 h. The absorbance of the samples was detected at the wavelength of 450 nm and 630 nm (reference wavelength). The cell viability ratio was calculated by A450–A630. Trypan blue exclusion method was used to test cell viability.

### 2.6. Measurement of Transcription by Real-Time Quantitative RT-PCR

The total RNA was isolated from cells by using a High Pure RNA Isolation Kit (purchased from Roche) and subsequently reverse-transcribed into cDNA by M-MLV reverse transcriptase (purchased from Promega). The expression (comparative transcription) of target gene mRNA was measured by quantitative RT-PCR and normalized by the household gene gapdh mRNA. The primer sequences used are as follows: 5′-Gag: ATCAAGCAGCCATGCAAATG; 3′-Gag: CTGAAGGGTACTAGTAGTTCC; 5′-Gapdh: TGGGTGTGAACCATGAGAAG; 3′-Gapdh: ATGGACTGTGGTCATGAGTC; 5′-HDAC10: ATGTGGCTGTTCGGAGAGGC; 3′-HDAC10: CTGCACTCCTGGCTGCAATG. The program is below: 95 °C, 10 min, 1 cycle; 95 °C 10 s, 55 °C or 60 °C 20 s, 72 °C 30 s, 40 cycles; 72 °C 2 min, 1 cycle.

### 2.7. Measurement of Total Viral DNA, Integrated DNA, and 2-LTR Viral DNA by Real-Time Quantitative PCR

Positively selected HDAC10 knockdown cells or scramble control cells were infected with HIV-1(N119) for 2 h and washed twice with PBS. After 12 h, cells were added with 10 μM AZT to restrict HIV-1 for only one round of replication. 24 h after infection, total DNA was isolated from cells using QIAmp blood DNA mini kit (Qiagen). The total levels of HIV-1 DNA, 2-LTR circles, and integrated DNA were quantified by Mx3000P real-time PCR system (Stratagene, CA) as described previously [35].

### 2.8. Luciferase Assay and Western Blot (WB)

Luciferase assay: After being infected with the progeny virus, TZMb1 cells were collected at the indicated time points and washed with PBS. The cell pellets were subjected to lysis buffer (purchased from Promega) for 10 min and the supernatant was collected. The luciferase activity in the supernatant was measured by a GLOMAX Luminometer (purchased from Promega) and normalized by the total protein concentration.

WB: Cells were lysed in RIPA buffer containing protease inhibitor cocktail and loaded onto an 8% or 10% SDS-PAGE gel. Then the protein was transferred onto the nitrocellulose membrane and incubated with specific primary antibody. The target protein expression was visualized by ECL substrate.

### 2.9. Co-Immunoprecipitation (Co-IP) Assay

The protocol for the co-IP assay studying the interaction between HIV-1 IN and cellular proteins was described in [36], with minor modifications. Briefly, 293T cells were transfected with GFP-INwt/mut and HDAC10-Myc or HDAC6-FLAG plasmids. After 48 h, cells were lysed with RIPA buffer supplemented with protease inhibitor for 30 min on the ice and the lysates were clarified by centrifugation (13,000 rpm for 30 min at 4 °C). Cell lysates were immunoprecipitated with rabbit anti-GFP or mouse anti-Myc antibody. The bound proteins were detected by a WB using anti-HDAC10, anti-HDAC6, or anti-GFP antibody, respectively. The interaction between HIV-1 IN-YFP and T7-Ku70 was detected by immunoprecipitation with rabbit anti-GFP followed by WB using anti-T7 to detect bound T7-Ku70 protein. For the interaction between HIV-1 IN-YFP and T7-LEDGF, transfected cells were lysed by CSK buffer (0.5% NP-40, 10 mMPipes pH 6.8, 10% (*w*/*v*) sucrose, 1 mM DTT, 1 mM MgCl_2_, 400 mM NaCl, and protease inhibitor) for 30 min on the ice. Cell lysates were immune-precipitated with rabbit anti-GFP antibody and followed by WB using anti-T7 to detect bound T7-LEDGF protein. Then, 2% of transfected cell lysates were used to detect the expression of different proteins by WB using corresponding antibodies.

### 2.10. Statistical Analysis

Statistical analysis of the results from the experiments, including luciferase assays, comparative quantitative RT-PCR, and p24 ELISA, were generated by the unpaired *t*-test (considered significant at *p* ≤ 0.05) or multiple *t*-test (with correction using the Holm-sidak method) with the assistance of GraphPad Prism 6.01 software.

## 3. Results

### 3.1. Endogenous HDAC10 Is Downregulated During HIV-1 Replication

**Figure 1:**
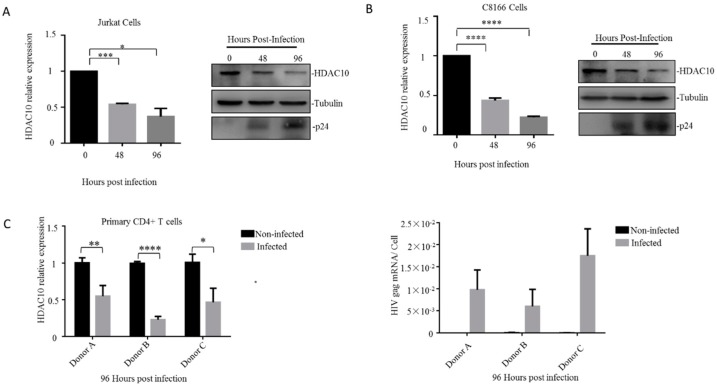
HDAC10 is downregulated in CD4^+^ T cells during HIV-1 infection. (**A**,**B**) The *HDAC10* mRNA (left panel) and protein (right panel) expression changes during HIV-1 infection in Jurkat cells (**A**) or C8166 cells (**B**). Jurkat cells or C8166 cells were infected with HIV-1 (N119) and collected at 0, 48, and 96 h. The *HDAC10* mRNA was detected by quantitative RT-PCR and normalized by house-keeping gene GAPDH while the HDAC10, tubulin, and HIV-1p24 proteins were detected by WB. (**C**) Primary CD4^+^ T cells isolated from three healthy individuals and each group has triplicates. healthy individuals were infected with HIV-1 (N119) and collected after 96 h of infection. The endogenous *HDAC10* mRNA (upper panel) was monitored by quantitative RT-PCR. HIV-1gag mRNA (lower panel) levels were detected by real-time RT-PCR and normalized by cell counts. Data are mean and sd (standard deviation). *, *p* < 0.05; **, *p* < 0.01, ***, *p* < 0.005; ****, *p* < 0.001. (Two-tailed unpaired *t*-test).

We first detected the endogenous HDAC10 expression changes during HIV-1 (N119 strain) infection in the Jurkat T lymphocytes. The cells were collected at 0, 48, and 96 h and the HDAC10 mRNA was detected by quantitative RT-PCR and normalized to the host gapdh gene (Figure 1A left panel). The HDAC10 protein was detected by WB analysis with an anti-HDAC10-specific antibody. HIV-1 infection was confirmed by detecting HIV-1 p24 protein in infected cells (Figure 1A right panel). The results showed that the HDAC10 comparative transcription was downregulated to 50% at 48 h and was further downregulated to 30% at 96 h (Figure 1A left panel). Consistent with the changes in HDAC10 mRNA, the HDAC10 protein level was downregulated to less than 50% at 96 h (Figure 1A right panel). We further confirmed this result in another CD4^+^ T lymphocyte cell line (C8166). Similarly, we found that HDAC10 mRNA and protein levels were also rapidly downregulated in HIV-1 infected C8166 cells (Figure 1B).

Next, we checked the HDAC10 expression changes upon HIV-1 infection in primary CD4^+^ T cells. Primary CD4^+^ T cells were isolated from three healthy individuals and subjected to HIV-1 infection. HIV-1 infection was confirmed by detecting HIV-1 gag mRNA expression (Figure 1C lower panel). The results showed that HDAC10 transcription was downregulated to 50%, 25%, and 40%, respectively, in different infected individual cells (Figure 1C upper panel). In summary, these results indicated that the transcription of HDAC10 is down-regulated during HIV-1 replication, which is consistent with our previous results [5].

### 3.2. HDAC10 Downregulation Benefits Viral Replication

**Figure 2:**
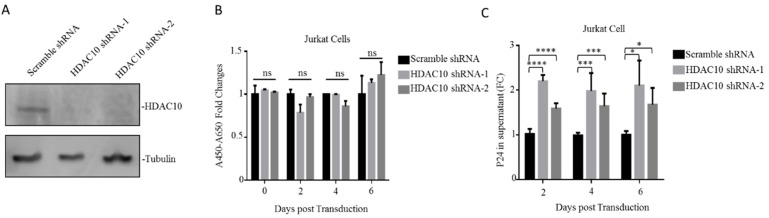
HDAC10 downregulation benefits HIV-1 replication. (**A**) The HDAC10 protein expression in HDAC10 shRNA-1, shRNA-2, and transduced cells or scramble shRNA transduced Jurkat cells. (**B**) The cell proliferation of HDAC10 shRNA or scramble shRNA transduced cells were detected by WST-1 assay on 2nd, 4th, and 6th day. (**C**) HDAC10 shRNA, or scramble shRNA transduced cells, were subjected to the infection by HIV-1 (N119) and the supernatants from infected cells were collected at indicated time point. The viral protein p24 in the supernatant was detected by anti-p24 ELISA. Data are mean and sd calculated from two duplicate samples. Ns, no significance; *, *p* < 0.05; ***, *p* < 0.005; ****, *p* < 0.001. (Two-tailed unpaired *t*-test).

After demonstrating the downregulation of HDAC10 during HIV-1 infection, we next investigated the effect of HDAC10 downregulation on HIV-1 infection and replication. First, HDAC10 knockdown (KD) cells were generated by RNA interference, as described in Materials and Methods. The WB result showed that HDAC10 protein was efficiently knocked down in Jurkat cells by HDAC10 shRNA-1 or shRNA-2 (Figure 2A). The WST-1 proliferation assays show that HDAC10 knockdown did not influence cell proliferation (Figure 2B).

We then evaluated the influence of HDAC10 downregulation on viral replication. Briefly, HDAC10-KD cells or control cells were infected with HIV-1 (N119) overnight and washed twice with PBS. The supernatant was collected at the indicated time points, and the production of virus in the supernatant was detected by anti-p24 ELISA. The results showed that compared with the control treated cells, HDAC10-KD cells produced a significantly higher level of p24 (Figure 2C), suggesting that HDAC10 downregulation enhances virus production.

### 3.3. The Downregulation of HDAC10 Facilitates Viral Integration

**Figure 3:**
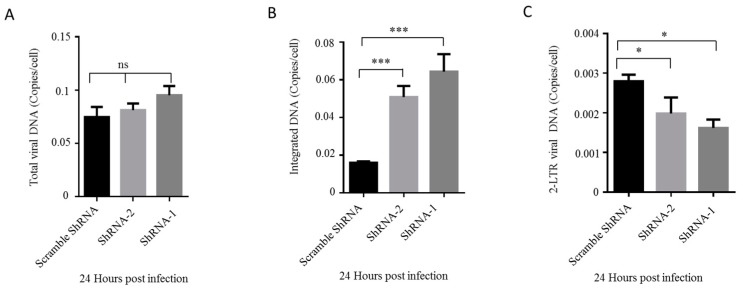
HDAC10 downregulation enhances viral integration. HDAC10 shRNA transduced or scramble shRNA transduced Jurkat cells were infected with HIV-1 (N119). 24 h later, cells were collected and total viral DNA (**A**), viral integrated DNA (**B**), and 2-LTR DNA (**C**) were analyzed by quantitative PCR. Data are mean and sd. Ns, no significance; *, *p* < 0.05; ***, *p* < 0.005. (Two-tailed unpaired *t*-test).

Upon demonstrating that HDAC10-KD benefits viral replication, we examined which step of replication is facilitated by HDAC10 downregulation. Briefly, we infected HDAC10-KD Jurkat cells and control cells with HIV-1 for 24 h and detected the total viral DNA, integrated viral DNA, and 2-LTR viral DNA in these cells by real-time quantitative PCR. The results showed that there is a slight increase in total viral DNA in HDAC10-KD cells when compared with control cells (Figure 3A). However, the integrated viral DNA increased 2–3-fold in HDAC10-KD cells (Figure 3B). Intriguingly, the levels of 2-LTR DNA decreased to 50%–70% in HDAC10-KD cells (Figure 3C) at 24 h post-infection, implying that the high level of integrated DNA was not due to an increased DNA nuclear import. Overall, these results indicated that HDAC10 downregulation enhances HIV-1 replication by promoting viral integration.

### 3.4. HDAC10 Interacts with HIV-1 IN Through Binding to the Region of Amino Acids 55 to 165 of IN

**Figure 4:**
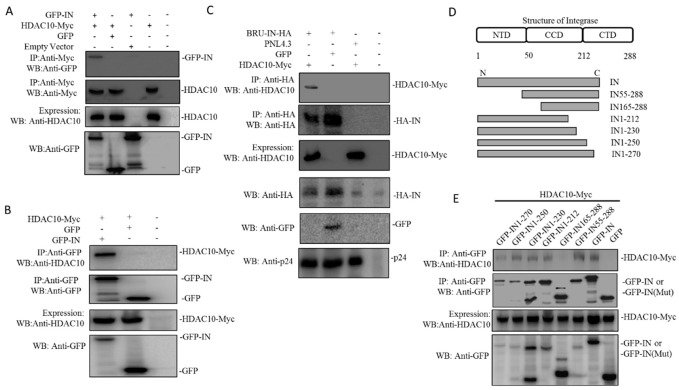
HDAC10 interacts with HIV-1 IN in co-transfected 293T cells. (**A**) GFP or GFP-IN was co-expressed with HDAC10-Myc in 293T cells for 48 h and cells were subjected to co-IP analysis by using anti-Myc antibody. Bound GFP-IN was detected by the anti-GFP antibody. (**B**) Above transfected 293T cells were subjected to co-IP by using rabbit anti-GFP antibody. Bound HDAC10 was detected by the anti-HDAC10 antibody. (**C**) HIV-1 Bru-IN-HA plasmid was co-expressed with HDAC10-Myc in 293T cells. Meanwhile, HIV-1PNL4.3 plus GFP plasmid was included as a control. Cells were subjected to co-IP analysis by using rabbit anti-HA antibody. Bound HDAC10 was detected by the anti-HDAC10 antibody. (**D**) The schematic of HIV-1 Integrase deletion mutant tagged with GFP. (**E**) GFP-IN wild type or its deletion mutant was co-expressed with HDAC10-Myc in 293T cells. Cells were subjected to Co-IP analysis by using anti-GFP antibody. Bound HDAC10 was detected by the anti-HDAC10 antibody. All the above expressions of HDAC10-Myc, GFP-INwt/mut, or IN-HA were detected by WB using anti-HDAC10, anti-GFP, or anti-HA antibody. Panels **A**–**E** show the presentative WB images from two independent experiments.

During the process of viral integration, HIV-1 IN is a key enzyme that catalyzes the insertion of the viral genome into host chromatin [37]. To investigate the underlying mechanisms by which HDAC10 downregulation enhances viral integration, we investigated the interaction between HIV-1 IN and HDAC10. Here, we examined the interaction of HDAC10 with HIV-1 IN using a co-IP assay. Briefly, 293T cells were co-transfected with HDAC10-Myc and GFP-IN or GFP. Meanwhile, GFP-IN or HDAC10-Myc was transfected alone as a control. After 48 h, the cell lysates were processed by co-IP using anti-Myc antibody. The pull-down protein was detected by WB using anti-GFP antibody. The results showed that only GFP-IN can detect in GFP-IN/HDAC10-myc IP samples, indicating HIV-1 IN interacts with HDAC10 (Figure 4A). The interaction between HDAC10 and HIV-1 IN was further confirmed by co-IP using anti-GFP antibody. As expected, WB using anti-HDAC10 can detect bound HDAC10 in HDAC10/GFP-IN co-IP sample, but not in HDAC10/GFP co-IP sample (Figure 4B). Finally, we confirmed the interaction between HDAC10 and HIV-1 IN by co-expression of the HIV-1 provirus plasmid Bru-IN-HA and HDAC10-Myc in 293T cells. Meanwhile, Bru-IN-HA and GFP, or HIV-1 PNL4.3 and HDAC10 were cotransfected as control. IN-HA was pulled down with an anti-HA antibody. The WB results revealed that bound HDAC10 can be detected in the Bru-IN-HA/HDAC10 co-IP sample, but not in PNL4.3/HDAC10 or Bru-IN-HA/GFP co-IP sample (Figure 4C).

After demonstrating the interaction between HDAC10 and HIV-1 IN, we further identified the region of IN that is essential for HDAC10 binding. A series of IN deletion mutants tagged with GFP, including GFP-IN55-288, GFP-IN165-288, GFP-IN1-212, GFP-IN1-230, GFP-IN1–250, and GFP-IN1–270 (Figure 4D), as described previously [38]. We co-transfected 293T cells with IN wild type or deletion mutant plasmid and HDAC10, and the cell lysates were processed by co-IP using anti-GFP antibody. We found that GFP and GFP-IN165-288 did not bind to HDAC10, whereas the wild type of GFP-IN and other truncated GFP-IN mutants (including GFP-IN 55–288, GFP-IN 1–212, GFP-IN 1–230, GFP-IN1-250, and GFP-IN1–270) still retained their HDAC10-binding ability, which suggested that IN 55–165 is essential for HDAC10 binding (Figure 4E).

### 3.5. The Presence of HDAC10 Weakens the Interaction of HIV-1 IN with LEDGF/p75

**Figure 5:**
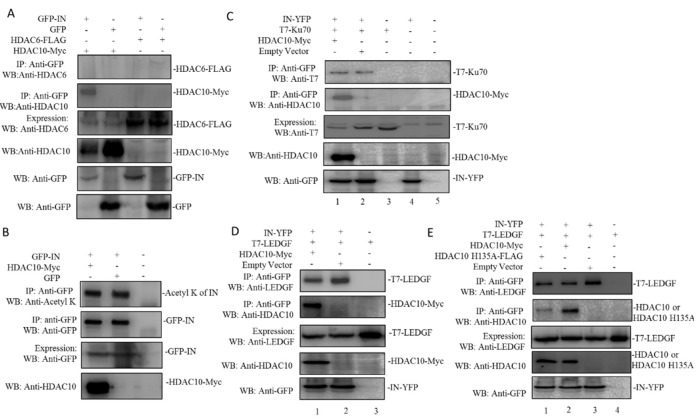
The effects of HDAC10 on the interaction between HIV-1 IN and cellular proteins LEDGF/p75 or KU70. (**A**) HIV-1 IN interacts with HDAC10 but not HDAC6. GFP or GFP tagged HIV-1 Integrase was co-expressed with HDAC10 or HDAC6 in 293T cells. Cells were subjected to co-IP using anti-GFP antibody and bound HDAC6 or HDAC10 was detected by anti-HDAC10 or anti-HDAC6 antibody. (**B**) The interaction of HDAC10 and HIV-1 Integrase does not change the lysine acetylation state of HIV-1 Integrase. GFP or GFP-IN and HDAC10 co-transfected 293T cells were subjected to co-IP analysis by using anti-GFP antibody and the bound GFP-IN was detected by anti-acetyl K antibody or anti-GFP antibody. (**C**) IN-YFP was co-expressed with T7-Ku70 and/or HDAC10-Myc in 293T cells. Cells were subjected to co-IP analysis by using anti-GFP antibody and bound T7-Ku70 or HDAC10-Myc was detected by WB using anti-T7 or anti-HDAC10 antibody. (**D**,**E**) IN-YFP was co-expressed with T7-LEDGF and /or HDAC10wt/H135A in 293T cells. Cells were subjected to co-IP analysis by using anti-GFP antibody and bound T7-LEDGF or HDAC10-Myc was detected by WB using anti-T7 or anti-HDAC10 antibody. All the above expressions of HDAC10-Myc, HDAC10-H135A, IN-YFP, T7-Ku70, or T7-LEDGF were detected by WB using anti-HDAC10, anti-GFP, or anti-T7 antibody. Panels **A**–**E** show the representative WB image from two independent experiments.

HDAC6 and HDAC10 belong to the HDAC class IIB family, and these proteins contain a unique, putative second catalytic domain that is not found in other HDACs [11]. We, therefore, further investigated whether HIV-1 IN also interacts with HDAC6. Briefly, we transfected 293T cells with GFP-IN with HDAC10 or HDAC6. Meanwhile, GFP was co-transfected with HDAC10 or HDAC6 as control. The co-IP assay showed that only HDAC10 can be pulled down by GFP-IN, but not HDAC6 (Figure 5A), indicating that HIV-1 IN specifically interacts with HDAC10.

All the above results showed that HDAC10 can specifically interact with HIV-1 IN by binding to IN 55–165, but how this interaction influences IN activity is still not clear. As an essential viral protein in viral integration, HIV-1 IN interacts with numerous cellular cofactors and exhibits multifunctional properties, which are tightly impacted by different posttranslational modifications [39,40]. The increase in the acetylation of HIV-1 IN can enhance the affinity of this enzyme for DNA and promote DNA strand transfer activity, which subsequently enhances viral integration [41,42]. Here, we examined whether HDAC10 can post-translationally modify the lysine acetylation state of IN. Briefly, GFP-IN was co-transfected with HDAC10 or with GFP plasmids (control) for 48 h. The GFP-IN protein was pulled down with an anti-GFP antibody. The acetylated lysine in HIV-1 IN was detected with an anti-acetylated lysine antibody. The results showed that in the presence or absence of HDAC10, the lysine acetylation state of HIV-1 IN was not significantly changed, which indicated that HDAC10 does not affect integration through regulating the lysine acetylation of IN (Figure 5B).

HIV-1 IN engages with different host cell proteins to exploit cellular machinery for an efficient viral integration [19]. For instance, Ku70, a DNA repair protein that is part of the nonhomologous end-joining (NHEJ) pathway, interacts with HIV-1 IN and protects IN from prosomal degradation [36,43,44,45]. Similarly, LEDGF, a cotranscription factor, has been reported to tether HIV-1 IN associated with the viral genome to the host chromatin and induce subsequent viral integration [46,47]. Here, we investigated whether HDAC10 regulates HIV-1 IN activity by affecting the interaction of HIV-1 IN with other cellular protein(s). Briefly, 293T cells were cotransfected with IN-YFP and T7-Ku70, with or without of HDAC10 plasmid. IN-YFP was pulled down with an anti-GFP antibody, and HDAC10 or Ku70 was detected with anti-HDAC10 or T7 antibody, respectively. As expected, the WB results showed that Ku70 can be detected in the cell lysis samples (Figure 5C, upper panel, lane 1, 2 and 3). However, the levels of bound Ku70 between IN-YFP/Ku70 sample and IN-YFP/Ku70/HDAC10 sample have no significant difference (Figure 5C). Similarly, we also investigated whether the presence of HDAC10 can influence the interaction between HIV-1 IN and LEDGF. Interestingly, the results showed that compared to the IN-YFP/LEDGF sample, the level of bound LEDGF was significantly decreased in presence of HDAC10 (Figure 5D, upper panel, lane 1 to 2). To examine whether the catalytic activity of HDAC10 affects the interaction between IN and LEDGF, we further tested the IN/LEDGF interaction in the presence of a catalytically inactive mutant of HDAC10 (HDAC10 H135A). As shown in Figure 5E, although the affinity of HDAC10 H135A to IN was lower than the wild type HDAC10 (upper panel, lane1 to 2), this mutant was still able to weaken the interaction of HIV-1 IN with LEDGF (upper panel, lane 1, 2, to 3).

### 3.6. The Downregulation of Endogenous HDAC10 Enhances the Infectiousness of Progeny Virus

**Figure 6:**
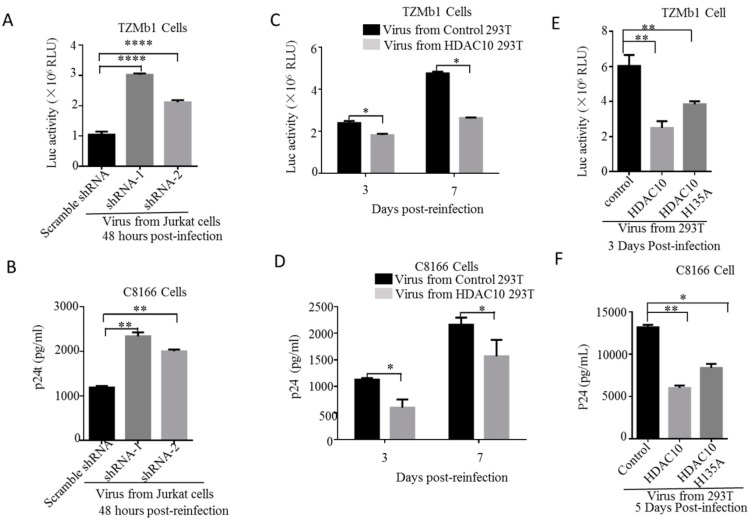
The infectivity of virus produced from HDAC10-KD or HDAC10 overexpressed cells. (**A**,**B**). The progeny virus was produced from HDAC10-KD Jurkat cells are used to infect TZMb1 and C8166 cells. The infectivity of virus was detected by measuring luciferase activity in TZMb1 cells (**A**) or p24 production in the supernatant from C8166 cells (**B**). (**C**,**D**) The progeny virus was produced from HIV-1 PNL4.3 provirus and HDAC10, or HIV-1 PNL4.3 provirus and GFP (control) co-transfected 293T cells. The same amount of progeny virus was used to infect TZMb1 or C8166 cells and the infection of the virus was detected by measuring luciferase activity in TZMb1 cells (**C**) or p24 in the supernatant from C8166 cells (**D**). (**E**,**F**) The infectivity of progeny viruses produced from PNL4.3/HDAC10, PNL4.3/HDAC10 H135A, or PNL4.3/GFP overexpressed cells. The viruses were produced from HIV-1 PNL4.3/HDAC10, PNL4.3/HDAC10H135A, or PNL4.3/GFP co-transfected 293T cells. The same amount of progeny virus was used to infect TZMb1 or C8166 cells and the infectivity of the virus was detected as described above. The values presented are the means and standard deviations from two independent experiments. *, *p* < 0.05; **, *p* < 0.01; ****, *p* < 0.001. (Two-tailed unpaired *t*-test).

We also investigated the effect of HDAC10 downregulation on the late stage of HIV-1 replication. Here, we first evaluated the infectiousness of the progeny virus from HDAC10-KD cells or control cells. Briefly, the same amount of progeny virus (adjusted by p24) from the HIV-1 infected HDAC10-KD Jurkat cells or HIV-1 infected control Jurkat cells was used to infect TZMb1 cells that express human CD4, CCR5, and CXCR4 and contain HIV-1 Tat-regulated reporter firefly luciferase genes [48]. The results showed that the relative firefly luciferase activity (Luc activity) of TZMb1 cells infected with the progeny virus from HDAC10-KD cells was 2–3 fold increased than the cells infected with progeny virus from control Jurkat cells. (Figure 6A). This observation was confirmed in C8166 CD4^+^ T lymphocyte cells. As expected, the p24 level of the supernatant of C8166 cells infected with progeny virus from HDAC10-KD cells was 2-fold higher than that from control cells (Figure 6B). Thus, the progeny virus from HDAC10-KD cells is more infectious than that from control cells.

Next, we tested whether HDAC10 overexpression influences the infectiousness of progeny virus. Briefly, we cotransfected 293T cells with HIV-1 pNL4.3 and HDAC10 plasmid or HIV-1 pNL4.3 and GFP plasmid (control). The generated viruses were used to infect TZMb1 or C8166 cells. The results showed that the progeny virus from HDAC10 overexpression cells induced less Luc activity in TZMb1 cells and produced less p24 in C8166 cells, which suggested that HDAC10 overexpression inhibited the progeny virus infectiousness (Figure 6C,D).

Upon demonstrating that the downregulation of HDAC10 enhances the infectiousness of progeny virus, we further investigated the impact of the enzymatic domain of HDAC10 on the infectiousness of the progeny virus. To this end, 293T cells were transfected with HIV-1 pNL4.3 and HDAC10, HIV-1 pNL4.3, and HDAC10H135A (catalytically inactive mutant) [10], or HIV-1 plasmid pNL4.3 and GFP (control). As expected, the virus from HDAC10-overexpressing cells produced less Luc activity in TZMb1 cells and less p24 in the supernatant from C8166 cells than that from control cells. However, the Luc activity and p24 produced by virus from HDAC10 H135A-overexpressing cells were higher than that from HDAC10-overexpressing (Figure 6E,F), indicating that the enzymatic domain of HDAC10 may be partially responsible for the ability of HDAC10 to inhibit progeny virus infectiousness.

## 4. Discussion

Although previous studies have identified a series of HDACs and characterized their roles in HIV-1 replication, our understanding of the influence of HDACs on HIV-1 replication is still limited. In this study, we identified HDAC10 as an HIV-1 inhibitory factor and studied its inhibitory function during HIV-1 replication. By real-time quantitative RT-PCR and WB, we found that HDAC10 is transcriptionally downregulated by HIV-1 in CD4^+^ T cells. We also revealed that HDAC10 downregulation promotes viral replication by enhancing viral integration. To investigate the underlying mechanism, we demonstrated that HDAC10 can interact with HIV-1 IN by using a cell-based co-immunoprecipitation assay. Deletion analysis revealed that HDAC10 interacts with HIV-1 IN by binding to the IN region encompassing 55–165 aa. Furthermore, our results showed that the interaction of HDAC10 with HIV-1 IN does not alter the lysine acetylation of HIV-1 IN but weakens the interaction between HIV-1 IN and LEDGF, which contributes to the inhibition of viral integration. In addition to affecting viral integration, our results also showed that the infectiousness of progeny virus is inversely correlated with the cellular HDAC10 level and that HDAC10 downregulation increased the infectiousness of progeny virus. Taken together, our study suggested that HDAC10 acts as an inhibitory factor against HIV-1 and that its downregulation is beneficial for both the early and late stages of HIV-1 replication.

Previous studies have identified several restriction factors and investigated the relevant HIV-1 evading mechanism [49]. For instance, A3G catalyzes the deamination of cytidine to uridine and causes the mutation of the viral genome, but A3G can be counteracted by the viral protein Vif [50]. BST-2 prevents the release of HIV-1 virions by tethering virions on the cell surface, but BST-2 can induce degradation by the viral protein Vpu [51]. Here, we report that the cellular protein HDAC10 is downregulated by HIV-1 during HIV-1 replication. Similar to A3G and BST-2, we found that HDAC10 levels decreased rapidly and were less than 50% at 48 h post infection. The downregulation of HDAC10 is not by proteasomal degradation, but by decreasing its mRNA expression. In our previous study, RNA-seq results showed that HIV-1 virion-associated envelope glycoprotein caused the downregulation of endogenous HDAC10. However, except for envelope glycoprotein, whether other viral proteins also directly contribute to this downregulation needs further investigation [5].

During the early stage of HIV-1 replication, HIV-1 integration is an essential step for HIV-1 replication [52]. HIV-1 IN is a key enzyme for this process, and this enzyme mainly mediates viral integration with the assistance of many cellular factors [40,53]. Some of these cellular factors, such as Ku70, can enhance integration, while other factors, such as KAP1, can inhibit this process [36,54]. Here, our study indicated a host cellular factor of HIV-1 IN, HDAC10, acts as an inhibitory factor in viral integration. In our results, we found that HDAC10 knockdown enhanced the viral integration without affecting total viral DNA synthesis. Interestingly, the knockdown of HDAC10 also slightly reduced 2-LTR DNA level, but the underlying mechanism is still elusive. It may be possible that HDAC10-KD may affect the balance of integrated DNA and 2-LTR DNA level in the nucleus by promoting viral integration and consequently decreasing the recombination of unintegrated DNA. Further, our results showed that HDAC10 downregulation enhances viral integration by interacting with the HIV-1 IN 55–165aa region. Based on the results, it is reasonable to propose that the interaction between HDAC10 and HIV-1 IN may modify the lysine acetylation of HIV-1 IN. Previous publications have reported that the acetylation state of the HIV-1 IN is associated with the activity of HIV-1 IN [41,42]. Acetylation increases IN affinity for DNA and promotes DNA strand transfer activity, which consequentially enhances viral integration [41]. However, our results showed that HDAC10 does not influence the lysine acetylation of HIV-1 IN, implying that HDAC10 may not perform its enzymatic function but rather performs other regulatory functions during viral integration [55]. Although HDAC10 does not alter the acetylation of HIV-1 IN, interestingly, we found that the presence of HDAC10 weakens the interaction of HIV-1 IN with LEDGF. The interaction of HIV-1 IN with LEDGF is essential for the process of viral integration. Previously, several groups have reported that the impaired interaction of HIV-1 IN with LEDGF can directly block viral integration [56,57,58]. Based on the above information, it is possible that HDAC10 weakens the interaction of HIV-1 IN with LEDGF, which consequently inhibits viral integration.

Finally, we also provided evidence that HDAC10 affects the late stage of viral replication. At the late stage of viral replication, many cellular factors can influence progeny virus assembly and maturation, which consequently regulates progeny virus infectiousness [59]. For instance, in addition to preventing the release of viral particles, BST-2 also impairs HIV-1 progeny virus infectiousness through the accumulation of pr55 Gag precursor and the p40 Gag intermediates, which leads to the loss of a mature core in the majority of HIV-1 particles [60]. In our study, we found that the level of cellular HDAC10 is inversely correlated with the infectiousness of progeny virus. The downregulation of HDAC10 can enhance progeny virus infectiousness, while the upregulation of HDAC10 can inhibit progeny virus infectiousness, which suggests that endogenous HDAC10 performs an inhibitory function in progeny virus infectiousness. Previous studies have reported that progeny virus infectiousness is associated with the interaction of endogenous LEDGF with HIV-1 IN in producer cells. The disruption of HIV-1 IN binding to endogenous LEDGF can significantly decrease progeny virus infectiousness [26,61]. For the underlying mechanism, Belete Ayele Desimmie et al. found that the disruption of the interaction between newly synthesized HIV-1 IN and endogenous LEDGF affects IN multimerization during virion assembly. They also found approximately 70% of the progeny virions do not form a core or display aberrant empty cores with a mis-localized electron dense ribonucleoprotein [62]. Therefore, it is possible that the presence of HDAC10 impairs the interaction of HIV-1 IN and endogenous LEDGF, which consequentially contributes to the loss of progeny viral infectiousness.

In summary, we showed that HDAC10 is a potent cellular factor that influences viral integration and progeny virus infectiousness. This study provides new insight into the interaction between cellular protein HDAC10 and viral protein IN, which offers a potential strategy for HIV-1 therapy.

## References

[1] Herbein G., Wendling D. (2010). Histone deacetylases in viral infections. Clin. Epigenetics.

[2] Shirakawa K., Chavez L., Hakre S., Calvanese V., Verdin E. (2013). Reactivation of latent hiv by histone deacetylase inhibitors. Trends Microbiol..

[3] Valera M.-S., de Armas-Rillo L., Barroso-González J., Ziglio S., Batisse J., Dubois N., Marrero-Hernández S., Borel S., García-Expósito L., Biard-Piechaczyk M. (2015). The hdac6/apobec3g complex regulates hiv-1 infectiveness by inducing vif autophagic degradation. Retrovirology.

[4] Liu Y., Peng L., Seto E., Huang S., Qiu Y. (2012). Modulation of histone deacetylase 6 (hdac6) nuclear import and tubulin deacetylase activity through acetylation. J. Biol. Chem..

[5] Ran X., Ao Z., Trajtman A., Xu W., Kobinger G., Keynan Y., Yao X. (2017). Hiv-1 envelope glycoprotein stimulates viral transcription and increases the infectivity of the progeny virus through the manipulation of cellular machinery. Sci. Rep..

[6] Tong J.J., Liu J., Bertos N.R., Yang X.-J. (2002). Identification of hdac10, a novel class ii human histone deacetylase containing a leucine-rich domain. Nucleic Acids Res..

[7] Kao H.-Y., Lee C.-H., Komarov A., Han C.C., Evans R.M. (2002). Isolation and characterization of mammalian hdac10, a novel histone deacetylase. J. Biol. Chem..

[8] Kotian S., Liyanarachchi S., Zelent A., Parvin J.D. (2011). Histone deacetylases 9 and 10 are required for homologous recombination. J. Biol. Chem..

[9] Oehme I., Linke J.-P., Böck B.C., Milde T., Lodrini M., Hartenstein B., Wiegand I., Eckert C., Roth W., Kool M. (2013). Histone deacetylase 10 promotes autophagy-mediated cell survival. Proc. Natl. Acad. Sci. USA.

[10] Li Y., Peng L., Seto E. (2015). Histone deacetylase 10 regulates the cell cycle g2/m phase transition via a novel let-7–hmga2–cyclin a2 pathway. Mol. Cell. Biol..

[11] Guardiola A.R., Yao T.-P. (2002). Molecular cloning and characterization of a novel histone deacetylase hdac10. J. Biol. Chem..

[12] Yang Y., Huang Y., Wang Z., Wang H.-T., Duan B., Ye D., Wang C., Jing R., Leng Y., Xi J. (2016). Hdac10 promotes lung cancer proliferation via akt phosphorylation. Oncotarget.

[13] Martínez F.P., Tang Q. (2013). Identification of cellular proteins that interact with human cytomegalovirus immediate-early protein 1 by protein array assay. Viruses.

[14] Craigie R. (2012). The molecular biology of hiv integrase. Future Virol..

[15] Al-Mawsawi L.Q., Sechi M., Neamati N. (2007). Single amino acid substitution in hiv-1 integrase catalytic core causes a dramatic shift in inhibitor selectivity. FEBS Lett..

[16] Chiu T.K., Davies D.R. (2004). Structure and function of hiv-1 integrase. Curr. Top. Med. Chem..

[17] Engelman A. (2007). Host cell factors and hiv-1 integration. Future HIV Ther..

[18] Craigie R., Bushman F.D. (2014). Host factors in retroviral integration and the selection of integration target sites. Microbiol. Spectr..

[19] Taltynov O., Desimmie B.A., Demeulemeester J., Christ F., Debyser Z. (2012). Cellular cofactors of lentiviral integrase: From target validation to drug discovery. Mol. Biol. Int..

[20] Parissi V., Calmels C., De Soultrait V.R., Caumont A., Fournier M., Chaignepain S., Litvak S. (2001). Functional interactions of human immunodeficiency virus type 1 integrase with human and yeast hsp60. J. Virol..

[21] Kessl J.J., Kutluay S.B., Townsend D., Rebensburg S., Slaughter A., Larue R.C., Shkriabai N., Bakouche N., Fuchs J.R., Bieniasz P.D. (2016). Hiv-1 integrase binds the viral rna genome and is essential during virion morphogenesis. Cell.

[22] Bukovsky A., Göttlinger H. (1996). Lack of integrase can markedly affect human immunodeficiency virus type 1 particle production in the presence of an active viral protease. J. Virol..

[23] Jurado K.A., Wang H., Slaughter A., Feng L., Kessl J.J., Koh Y., Wang W., Ballandras-Colas A., Patel P.A., Fuchs J.R. (2013). Allosteric integrase inhibitor potency is determined through the inhibition of hiv-1 particle maturation. Proc. Natl. Acad. Sci. USA.

[24] Dar M.J., Monel B., Krishnan L., Shun M.-C., Di Nunzio F., Helland D.E., Engelman A. (2009). Biochemical and virological analysis of the 18-residue c-terminal tail of hiv-1 integrase. Retrovirology.

[25] Engelman A. (1999). In vivo analysis of retroviral integrase structure and function. Adv. Virus Res..

[26] Christ F., Shaw S., Demeulemeester J., Desimmie B.A., Marchand A., Butler S., Smets W., Chaltin P., Westby M., Debyser Z. (2012). Small molecule inhibitors of the ledgf/p75 binding site of integrase (ledgins) block hiv replication and modulate integrase multimerization. Antimicrob. Agents Chemother..

[27] Jayappa K.D., Ao Z., Wang X., Mouland A.J., Shekhar S., Yang X., Yao X. (2015). Human immunodeficiency virus type 1 employs the cellular dynein light chain 1 protein for reverse transcription through interaction with its integrase protein. J. Virol..

[28] Yao X.-J., Mouland A.J., Subbramanian R.A., Forget J., Rougeau N., Bergeron D., Cohen E.A. (1998). Vpr stimulates viral expression and induces cell killing in human immunodeficiency virus type 1-infected dividing jurkat t cells. J. Virol..

[29] Li S., Liu C., Klimov A., Subbarao K., Perdue M.L., Mo D., Ji Y., Woods L., Hietala S., Bryant M. (1999). Recombinant influenza a virus vaccines for the pathogenic human a/hong kong/97 (h5n1) viruses. J. Infect. Dis..

[30] Wahl-Jensen V., Kurz S.K., Hazelton P.R., Schnittler H.-J., Ströher U., Burton D.R., Feldmann H. (2005). Role of ebola virus secreted glycoproteins and virus-like particles in activation of human macrophages. J. Virol..

[31] Fischle W., Emiliani S., Hendzel M.J., Nagase T., Nomura N., Voelter W., Verdin E. (1999). A new family of human histone deacetylases related tosaccharomyces cerevisiae hda1p. J. Biol. Chem..

[32] Ao Z., Jayappa K.D., Wang B., Zheng Y., Kung S., Rassart E., Depping R., Kohler M., Cohen E.A., Yao X. (2010). Importin α3 interacts with hiv-1 integrase and contributes to hiv-1 nuclear import and replication. J. Virol..

[33] Ao Z., Huang G., Yao H., Xu Z., Labine M., Cochrane A.W., Yao X. (2007). Interaction of human immunodeficiency virus type 1 integrase with cellular nuclear import receptor importin 7 and its impact on viral replication. J. Biol. Chem..

[34] Ao Z., Zhu R., Tan X., Liu L., Chen L., Liu S., Yao X. (2016). Activation of hiv-1 expression in latently infected cd4^+^ t cells by the small molecule pkc412. Virol. J..

[35] Ao Z., Jayappa K.D., Wang B., Zheng Y., Wang X., Peng J., Yao X. (2012). Contribution of host nucleoporin 62 in hiv-1 integrase chromatin association and viral DNA integration. J. Biol. Chem..

[36] Zheng Y., Ao Z., Wang B., Jayappa K.D., Yao X. (2011). Host protein ku70 binds and protects hiv-1 integrase from proteasomal degradation and is required for hiv replication. J. Biol. Chem..

[37] Craigie R. (2001). Hiv integrase, a brief overview from chemistry to therapeutics. J. Biol. Chem..

[38] Zheng Y., Ao Z., Jayappa K.D., Yao X. (2010). Characterization of the hiv-1 integrase chromatin-and ledgf/p75-binding abilities by mutagenic analysis within the catalytic core domain of integrase. Virol. J..

[39] Chen H., Wei S.-Q., Engelman A. (1999). Multiple integrase functions are required to form the native structure of the human immunodeficiency virus type i intasome. J. Biol. Chem..

[40] Zheng Y., Yao X. (2013). Posttranslational modifications of hiv-1 integrase by various cellular proteins during viral replication. Viruses.

[41] Cereseto A., Manganaro L., Gutierrez M.I., Terreni M., Fittipaldi A., Lusic M., Marcello A., Giacca M. (2005). Acetylation of hiv-1 integrase by p300 regulates viral integration. EMBO J..

[42] Terreni M., Valentini P., Liverani V., Gutierrez M.I., Di Primio C., Di Fenza A., Tozzini V., Allouch A., Albanese A., Giacca M. (2010). Gcn5-dependent acetylation of hiv-1 integrase enhances viral integration. Retrovirology.

[43] Tuteja R., Tuteja N. (2000). Ku autoantigen: A multifunctional DNA-binding protein. Crit. Rev. Biochem. Mol. Biol..

[44] Downs J.A., Jackson S.P. (2004). A means to a DNA end: The many roles of ku. Nat. Rev. Mol. Cell Biol..

[45] Giffin W., Torrance H., Rodda D.J., Préfontaine G.G., Pope L., Haché R.J. (1996). Sequence-specific DNA binding by ku autoantigen and its effects on transcription. Nature.

[46] Cherepanov P., Maertens G., Proost P., Devreese B., Van Beeumen J., Engelborghs Y., De Clercq E., Debyser Z. (2003). Hiv-1 integrase forms stable tetramers and associates with ledgf/p75 protein in human cells. J. Biol. Chem..

[47] Hare S., Cherepanov P. (2009). The interaction between lentiviral integrase and ledgf: Structural and functional insights. Viruses.

[48] Takeuchi Y., McClure M.O., Pizzato M. (2008). Identification of gammaretroviruses constitutively released from cell lines used for human immunodeficiency virus research. J. Virol..

[49] Malim M.H., Bieniasz P.D. (2012). Hiv restriction factors and mechanisms of evasion. Cold Spring Harb. Perspect. Med..

[50] He Z., Zhang W., Chen G., Xu R., Yu X.-F. (2008). Characterization of conserved motifs in hiv-1 vif required for apobec3g and apobec3f interaction. J. Mol. Biol..

[51] Van Damme N., Goff D., Katsura C., Jorgenson R.L., Mitchell R., Johnson M.C., Stephens E.B., Guatelli J. (2008). The interferon-induced protein bst-2 restricts hiv-1 release and is downregulated from the cell surface by the viral vpu protein. Cell Host Microbe.

[52] Marchand C., Johnson A.A., Semenova E., Pommier Y. (2006). Mechanisms and inhibition of hiv integration. Drug Discov. Today Dis. Mech..

[53] Van Maele B., Busschots K., Vandekerckhove L., Christ F., Debyser Z. (2006). Cellular co-factors of hiv-1 integration. Trends Biochem. Sci..

[54] Allouch A., Di Primio C., Alpi E., Lusic M., Arosio D., Giacca M., Cereseto A. (2011). The trim family protein kap1 inhibits hiv-1 integration. Cell Host Microbe.

[55] Hai Y., Shinsky S.A., Porter N.J., Christianson D.W. (2017). Histone deacetylase 10 structure and molecular function as a polyamine deacetylase. Nat. Commun..

[56] Poeschla E.M. (2008). Integrase, ledgf/p75 and hiv replication. Cell. Mol. Life Sci..

[57] Llano M., Saenz D.T., Meehan A., Wongthida P., Peretz M., Walker W.H., Teo W., Poeschla E.M. (2006). An essential role for ledgf/p75 in hiv integration. Science.

[58] Ciuffi A., Llano M., Poeschla E., Hoffmann C., Leipzig J., Shinn P., Ecker J.R., Bushman F. (2005). A role for ledgf/p75 in targeting hiv DNA integration. Nat. Med..

[59] Lever A.M., Jeang K.-T. (2011). Insights into cellular factors that regulate hiv-1 replication in human cells. Biochemistry.

[60] Zhang J., Liang C. (2010). Bst-2 diminishes hiv-1 infectivity. J. Virol..

[61] Le Rouzic E., Bonnard D., Chasset S., Bruneau J.-M., Chevreuil F., Le Strat F., Nguyen J., Beauvoir R., Amadori C., Brias J. (2013). Dual inhibition of hiv-1 replication by integrase-ledgf allosteric inhibitors is predominant at the post-integration stage. Retrovirology.

[62] Desimmie B.A., Schrijvers R., Demeulemeester J., Borrenberghs D., Weydert C., Thys W., Vets S., Van Remoortel B., Hofkens J., De Rijck J. (2013). Ledgins inhibit late stage hiv-1 replication by modulating integrase multimerization in the virions. Retrovirology.

